# Dolosigranulum pigrum: Predicting Severity of Infection

**DOI:** 10.7759/cureus.9770

**Published:** 2020-08-15

**Authors:** John Sherret, Bhavesh Gajjar, Lamis Ibrahim, Ahmed Mohamed Ahmed, Utsab R Panta

**Affiliations:** 1 Internal Medicine, East Tennessee State University Quillen College of Medicine, Johnson City, USA; 2 Infectious Disease, East Tennessee State University Quillen College of Medicine, Johnson City, USA

**Keywords:** dolosigranulum pigrum, antibiotic susceptibility, opportunistic infection, immunocompromised

## Abstract

In this report, we describe a case of a 61-year-old male patient who had the bacterium *Dolosigranulum pigrum* growing in a blood culture. It was susceptible to ampicillin, ceftriaxone, levofloxacin, and vancomycin but was intermediately resistant to erythromycin. The patient did not have a negative outcome as a consequence of this bacterium, which retrospectively could have been predicted based on the epidemiological data within the patient's profile.

## Introduction

*Dolosigranulum pigrum* (*D. pigrum*) was first reported in 1993 when 16S RNA gene sequencing revealed previously uncharacterized gram-positive catalase-negative bacteria [1]. Since discovery the reporting of this bacterium has been increasing. While this increased incidence could be due to increased usage of RNA gene sequencing in clinical laboratories, it also could mark the emergence of an opportunistic infection particularly in immunocompromised patients [2]. Otherwise, it has been suggested that *D. pigrum* remains a commensal organism [2-5].

*D. pigrum* can cause eye infections, sepsis, nosocomial pneumonia, and ventilator-associated pneumonia [2,6,7]. It has been suspected in synovitis and cholecystitis and has also been isolated from gastric contents, blood and urine cultures, the nasopharyngeal regions, and even in the spinal cord [6,8,9]. *D. pigrum* has been reported in patients as young as two months and as old as 85 years [6]. Currently, 27 strains have been identified [10]. All strains are susceptible to beta-lactam antibiotics, and approximately half of the strains are known to be resistant to erythromycin [2,6]. In this report, we present a sighting of this bacterium, its antibiotic susceptibilities, and the risk factors that predict illness.

## Case presentation

Our patient was a 61-year-old male who presented to the emergency room for shortness of breath. He was hypoxic, requiring two liters of oxygen but his other vital signs were within a normal range. He also complained of bilateral leg swelling but denied fever, chills, cough, wheezing, palpitations, and dizziness. He was a former smoker and denied alcohol or illicit drug use. The patient had recently completed six weeks of cefepime and metronidazole for right foot osteomyelitis. He had a past medical history of heart failure with reduced ejection fraction, end-stage renal disease on hemodialysis, type II diabetes, asthma, stroke, and coronary artery disease with prior myocardial infarction and percutaneous intervention. Chest and foot X-rays did not show any concerns for infection.

He was treated for an acute exacerbation of congestive heart failure. He was given a one-time dose of cefepime in the emergency department and was continued on hemodialysis. Two days into admission, the blood culture revealed non-specific gram-positive organisms for which vancomycin was started. Five days later, the microbe was identified as *D. pigrum* and repeat blood cultures obtained six days after admission were negative. The susceptibilities showed *D. pigrum* to be pan-sensitive except for erythromycin which demonstrated intermediate resistance (Table 1). 

**Table 1:** Antibiotic Susceptibility of Dolosigranulum pigrum The bacterium was pan-sensitive with the exception of intermediate resistance that was demonstrated by erythromycin.  The symbol "S" represents antibiotics to which the bacterium was susceptible, while the symbol "I" represents antibiotics to which the bacterium was intermediately resistant.

Antibiotic	Susceptibility
Ampicillin	0.016 S
Ceftriaxone	0.016 S
Erythromycin	0.5 I
Levofloxacin	0.094 S
Vancomycin	0.125 S

## Discussion

The understanding of the general pathology and epidemiology of *D. pigrum *has been growing over time. Many studies have documented asymptomatic *D. pigrum* residency in the upper respiratory tracts [11-13]. However, several case reports to date have documented a variety of infections. One report mentions a 64-year-old male with rheumatoid arthritis who was taking chronic prednisone and methotrexate to control synovitis. His left arm became very tender. Blood cultures were drawn, antibiotics were started, and then an arthrocentesis was performed. While the blood cultures were positive for *D. pigrum*, the synovial fluid was sterile but had numerous amounts of leukocytes and followed an infectious pattern [8]. Another report is of a 73-year-old male with a medical history of chronic obstructive pulmonary disease due to heavy smoking, thrombocythemia treated with hydroxycarbamide, and childhood pulmonary tuberculosis who presented for acute respiratory insufficiency due to pulmonary embolism. Thirteen days into admission, the patient developed fever and blood cultures were drawn revealing *D. pigrum*. Two days later, he developed septic shock requiring emergent intubation and bronchoalveolar lavage which was positive for *Staphylococcus aureus* and *D. pigrum* [2]. In a third report, 25 patients with cystic fibrosis who were hospitalized provided sputum samples which detected *D. pigrum* and others [14]. Given the current knowledge that pulmonary infections in cystic fibrosis are polymicrobial, such detections are significant [14-18]. In a fourth report, a 51-year-old male with no past medical history was admitted for severe aneurysmal subarachnoid hemorrhage with respiratory distress and was subsequently intubated. On ventilator day 10, he developed signs of pneumonia and cultures of bronchial secretions ultimately revealed *D. pigrum* [7]. In a fifth report, *D. pigrum* was suspected to be the cause of an acute cholecystitis in a 76-year-old male with gallstones and no other past medical history. The blood cultures were positive for *D. pigrum* [9]. In a sixth report, the Centers for Disease Control and Prevention (CDC) reported on 27 isolates they had received. Twelve had come from blood cultures, nearly half of which had sepsis. Six were from the eye, four were from the nasopharynx, one was from the sinus, one was from sputum, and one was from the stomach suggesting an upper respiratory habitat for the bacterium [6]. Many of these patients were either less than 2 years of age or older than 65 years of age. In a seventh report, a 71-year-old female, an 85-year-old male, and a 78-year-old female all had keratitis with corneal specimens positive for *D. pigrum* [4]. This further suggests that *D. pigrum* is an emerging pathogen that particularly targets the immunocompromised and the elderly. 

In comparison to prior reports, our patient followed patterns that predicted a favorable outcome [2-9,11-15]. He was a 61-year-old male with no chronic immunosuppressive conditions or medications apart from the end-stage renal disease on dialysis due to type II diabetes. He was less than 65 years of age. Therefore, he was unlikely to experience septic shock or severe respiratory distress from this organism. While he presented with shortness of breath, it was not severe as the pulse saturation was 99% on two liters of oxygen. Only one of the blood cultures initially revealed a gram-positive organism which was treated with vancomycin. A transthoracic echocardiogram was negative for endocarditis. On day 6, the initial blood culture identified the organism as *D. pigrum*. Two repeat peripheral cultures and a culture obtained from the dialysis ash catheter were negative. The patient was afebrile and never exhibited a leukocytosis. Given that the patient was immunocompetent and younger than 65 years, he was unlikely to experience a serious infection. Future cases would be wise to consider these criteria in *D. pigrum* infections.

The bacterium *D. pigrum* is mostly pan-sensitive to conventional antibiotics. Our specimen was sensitive to beta-lactams, cephalosporin, fluoroquinolones, and vancomycin. However, it was intermediately resistant to erythromycin, which largely matched the CDC data [4,6-9]. The CDC data showed that 52% of their isolates were resistant to erythromycin, with most of those resistant samples appearing in corneal collections [6]. Despite our patient being on six weeks of cefepime and metronidazole for right foot osteomyelitis, the *D. pigrum* was pan-sensitive. These observations suggest that *D. pigrum* does not contain widespread antibiotic resistance.

## Conclusions

*D. pigrum* is a potential emerging opportunistic infection that favors those >65 and <2 years of age, the immunocompromised, and those with baseline pulmonary diseases, such as cystic fibrosis and severe chronic obstructive pulmonary disease. *D. pigrum* appears to be very susceptible to current antibiotics. It can be challenging to identify in the laboratory setting which can cause delays in obtaining results. Further research is required to obtain a more accurate incidence and rapid diagnosis of the bacterium. 
